# State-Level Variation in Abortion Stigma and Women and Men’s Abortion Underreporting in the USA

**DOI:** 10.1007/s11113-021-09657-4

**Published:** 2021-05-20

**Authors:** Isaac Maddow-Zimet, Laura D. Lindberg, Kate Castle

**Affiliations:** 1 Guttmacher Institute, New York, NY, USA; 2 Department of Sociology, University of Michigan, Ann Arbor, MI, USA

**Keywords:** Abortion, Measurement, Data quality, NSFG, Stigma

## Abstract

Abortion is highly stigmatized in most settings and severely underreported in demographic surveys. In the USA, variation in the context of abortion between states may influence respondents’ exposure to abortion stigma and create geographic variation in their likelihood of disclosing abortion in surveys. We used restricted geographic data from the 2006–2010 and 2011–2015 National Survey of Family Growth (NSFG) to investigate the association between abortion reporting in the USA and state-level structural factors that may influence respondents’ experience of abortion stigma. At the aggregate level, we compared the weighted number of abortions women reported in the NSFG to abortion counts derived from abortion provider censuses and test for variation in underreporting by state-level structural measures. At the individual level, we tested if state-level structural factors were associated with less reporting of abortion in the face-to-face (FTF) survey mode than the more confidential audio computer-assisted self-interviewing mode (ACASI) of the NSFG. We found that at the aggregate level, there were no differences in reporting by the state-level measures. At the individual level, about 40% of women and men who reported an abortion in their ACASI did not fully report in the FTF interview; however, there were few differences by any state-level factors. This study documents that abortion stigma plagues the quality of reporting in the USA for both women and men, regardless of which state they live in. Survey improvements to reduce abortion underreporting are needed.

## Introduction

Underreporting of abortion compromises the quality of individual-level demographic data in surveys in Europe, the USA, and the Global South (Houzard et al., 2000; Jones & Kost, 2007; Lindberg et al., 2020; MacQuarrie et al., 2018; Scott et al., 2019). It is well established that respondents may not report sensitive behaviors to provide more socially desirable responses (Tourangeau & Yan, 2007); with abortion, the sensitivity of the behavior is assumed to reflect high levels of abortion stigma in most settings. Kumar et al. (2009) characterize abortion stigma as “a negative attribute ascribed to women who seek to terminate a pregnancy that marks them, internally or externally, as inferior to ideals of womanhood”. Norris et al. (2011) highlight other causes of abortion stigma, including legal restrictions on the procedure, views of abortion as ‘dirty’ or ‘unhealthy’, and the intentional use of ostracizing behaviors by groups of people with religious or ethical oppositions to abortion.

Prior literature on abortion stigma distinguished between internalized and perceived stigma at the individual level (how women feel about their own abortion and the reactions they expect from others) and enacted stigma at the interpersonal level (how others treat them post disclosure) (Hanschmidt et al., 2016). Research has given less attention to structural factors in women’s environments that may influence their experiences of stigma. Yet broader work on stigma conceptualize it as a multilevel process, with structural stigma defined as “societal-level conditions, cultural norms, and institutional policies that constrain the opportunities, resources, and well-being of the stigmatized,” (Hatzenbuehler & Link, 2014) playing an important role.

Likewise, most research on abortion underreporting in the USA has focused on either survey-level design factors or individual-level characteristics associated with underreporting and have not addressed the potential influence of structural stigma (Cowan et al., 2016; Lindberg & Scott, 2018; Lindberg et al., 2020; Peytchev et al., 2010; Tennekoon, 2017; Tierney, 2019). However, the context in which respondents live also may influence their likelihood of disclosing abortion experiences. Within the USA, respondents living in different states are exposed to different structural factors that influence laws and policies, the visibility of abortion, and relevant socio-cultural attitudes. For example, the extent to which abortion is officially sanctioned or marginalized through abortion laws differs across states; more restrictive laws may reinforce the status of abortion as stigmatized behavior. State differences in religiosity and in attitudes toward abortion may influence how respondents perceive others will react to their disclosure (and thus the likelihood that they will disclose (Cowan, 2014). Additionally, the visibility of abortion may influence reporting, as women with less exposure to others with abortion experiences may feel it is a rarer event and more stigmatized (Kumar et al., 2009).

In this study, we use restricted geographic data from the National Survey of Family Growth (NSFG), a key demographic survey in the USA, to investigate the association between abortion reporting and a set of state-level measures of structural abortion stigma. We test if abortion reporting is less complete among respondents living in states with greater structural stigma. We also test if individuals exposed to greater structural abortion stigma in their state are less likely to report their abortion in the face-to-face (FTF) portion of the interview (where respondents may feel more pressure to provide socially desirable responses) than in the more confidential audio computer-assisted self-interview (ACASI) portion; ACASI was designed to improve reporting of sensitive behaviors and has been shown to elicit higher reports of abortion than the FTF interview (Lindberg & Scott, 2018; Turner et al., 1998). We also explore for the first time abortion underreporting among men in the USA. This analysis can help to identify ways in which survey questions about people’s reproductive histories can be improved to produce more accurate and complete data.

## Methods

### Data

We analyzed data from the NSFG, a nationally representative household survey of the non-institutionalized civilian population of US women and men^1^ aged 15–44 in the USA (Lepkowski et al., 2010). We pooled data from the 2006–2010 and 2011–2015 waves for a total sample size of 23,579 women and 19,724 men; we then used restricted data on respondents’ state of residence, accessed through the National Center for Health Statistics Research Data Center, to merge the pooled data with state-level measures of abortion stigma developed for this analysis (described below).

The NSFG asked all respondents to report pregnancies first in the FTF interview and then again in the ACASI portion. In the FTF interview, female respondents were asked how many times they have ever been pregnant, followed by detailed questions about each pregnancy, including the pregnancy outcome. In the ACASI they were asked separately their number of live births, abortions and miscarriages within the last five calendar years. Both the FTF and ACASI modes asked male respondents the total number of pregnancies they had ever fathered that did not end in a live birth and the outcome of those pregnancies.

### Measures

#### Structural Stigma

Informed by theoretical literature, we developed five proxy measures of different aspects of structural stigma (identified in italics below). First, we used a variable of the state *policy environment*, constructed in previous work, that characterized states as hostile to abortion, supportive, or ‘middle-ground’ based on the number of restrictions in the state in 2011 (Gold & Nash, 2012).

We also obtained proxy measures of the visibility of abortion within the state, which could influence how common or accepted respondents feel the procedure is: the *abortion rate* per 1000 women of reproductive age by state of residence (averaged across 2008 and 2013) and the *number of abortion clinics* per 1000 women of reproductive age (averaged across 2008 and 2014, and defined as facilities providing 400 or more abortions per year, which provide the large majority of abortions in the USA, and which are likely the most visible to respondents in our sample; Jones & Jerman, 2017).

We used data from the 2014 Religious Landscape Study to construct two distinct measures of the state cultural environment that may influence abortion stigma (Pew Research Center, 2016). First, to measure *public opinion* about abortion we categorized states by the proportion of respondents that believed abortion should be legal in “all or most cases.” The second was a measure of state *religiosity* (the proportion of respondents that said religion was “very important” in their life); while not all religions proscribe abortion, the links between religious belief and abortion stigma are well-documented (Frohwirth et al., 2018), and it provides a reasonable proxy measure for religion-based stigma.

### Individual Sociodemographics

The individual-level analysis controlled for sociodemographic characteristics measured in the NSFG that were associated with abortion underreporting in prior studies: age at time of interview (15–24, 25–29, 30–34, 35 +), race and Hispanic ethnicity (non-Hispanic white, non-Hispanic black, non-Hispanic other, Hispanic), parity (0 or 1 + prior births), household income at time of interview (0–99% of the federal poverty line, 100–299%, and 300% +), a dichotomous measure of urban residence, informal marital status (married, cohabiting, not in union), nativity (US-born or foreign-born), and a dichotomous indicator of whether the respondent said religion was very important in their life.

### Analytical Approach

We conducted two interrelated analyses of abortion reporting in the NSFG. The first, among female respondents only, compares weighted aggregate reports of abortions within specific groupings of states with external abortion counts by state of residence derived from censuses of abortion providers in the USA^2^ (Guttmacher Institute, 2018; Jones & Jerman, 2017); the latter are considered the most complete abortion counts available, as not all states report data to the Centers for Disease Control and Prevention (Jatlaoui, 2018).

We grouped states using each of the five proxy measures of abortion stigma (policy environment, abortion rate, number of abortion clinics, public opinion and state religiosity). For each except the first measure, we categorized states into low, middle and high terciles, so that a third of states fell into each category (see Table 1).

Within each state tercile group, we compared the number of abortions in the five years prior to the interview that women reported in the FTF interview with external estimates of the number of abortions obtained by residents of those same pooled states. We adjusted these external estimates to match the NSFG’s sampling frame and five year recall period following an approach used previously for national estimates (Lindberg et al., 2020). Our outcome measure—the level of underreporting—was the proportion of abortions reported in the NSFG as compared to external counts in each state tercile group. We assessed statistical significance on the basis of non-overlapping confidence intervals between state groups, a relatively conservative approach.

We complemented this aggregate analysis with an individual-level analysis of discrepancies in abortion reporting between the FTF and ACASI portions of the questionnaire. Among women and men reporting any abortions in the ACASI portion of the NSFG (men = 2442, women = 1408), we identified if respondents had reported fewer abortions in the FTF interview.For female respondents, we restricted the FTF counts to abortions ending in the five calendar years prior to the interview to parallel ACASI reports, including a buffer period of 6 months on either side to account for slight misdating; for male respondents the counts referred to abortions over their lifetime.^3^ For each gender we estimated separate logistic regressions of the association of reduced abortion reporting in the FTF than ACASI mode with each of the five structural stigma measures for the state in which they reside.^4^ An advantage of this individual-level approach (as compared to the aggregate analysis) is that it allows controls for demographic characteristics known to be associated with abortion reporting; it also allows us to examine underreporting among men, for whom there is no reliable external count of abortions. All analyses accounted for the complex survey design of the NSFG using the *svy* commands in Stata 15.1 (Stata-Corp 2017). Note that due to data restrictions, we are unable to include unweighted sample sizes for specific geographic groupings in the tables presented.

## Results

### Demographic Characteristic of Respondents

Table 1 describes the weighted distribution of respondents in each pooled geographic area, stratified by gender. The majority of both men and women reside in states with hostile abortion laws (58% and 59%, respectively), and in the states with abortion rates in the highest tercile (51% and 50%), while substantial pluralities live in states in the middle tercile of clinics per capita (40% and 40%), and of opinions about abortion legality (40% and 39%). Both male and female respondents are almost evenly distributed across states in the low, middle and high terciles of religiosity.

### Aggregate Analysis

**Fig. 1:**
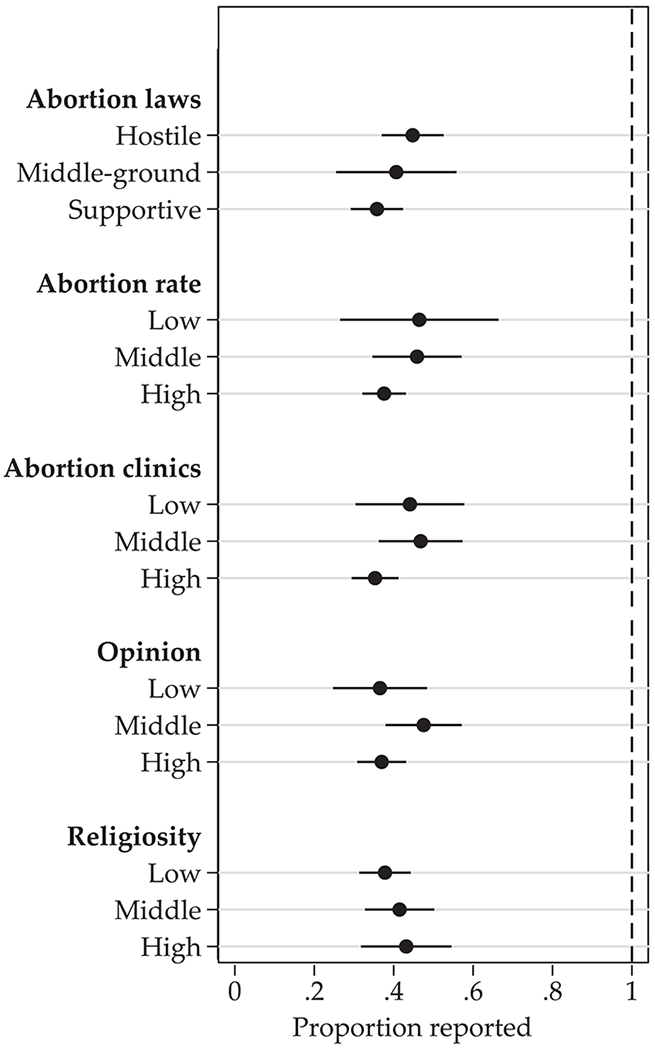
Proportion of abortions reported as compared to adjusted external counts, by measures of state-level abortion stigma, among women aged 15–44, pooled NSFG 2006–2015

Figure 1 shows the proportion of abortions reported by women in the FTF portion of the NSFG as compared to external counts with their associated 95% confidence intervals, stratified by our measures of states’ levels of abortion stigma. Abortion was severely underreported in every grouping, with NSFG weighted counts representing 35–48% of the abortions in the adjusted external count. There were no significant differences between any of the terciles within any measure of abortion stigma.

### Individual-Level Analysis

Tables 2 and 3 show the results of logistic regressions predicting reporting fewer abortions in the FTF than the ACASI interview among women and men reporting abortions in the ACASI. 41% of women (Table 2) and 39% of men (Table 3) reporting abortions in the ACASI reported fewer (or no) abortions in the FTF interview. Despite this high level of discordance in reporting, we find limited evidence of variation by the contextual measures of abortion stigma. For male respondents, no measure of state-level abortion stigma was associated with the odds of reporting fewer abortions in the FTF interview than in the ACASI. Among female respondents, living in a state with higher number of abortion clinics per capita (AOR = 0.50 for middle, and AOR = 0.57 for high) or states where greater proportions of the population believed abortion should be legal in most or all cases (AOR = 0.66) was associated with lower odds of giving a discordant report in the FTF interview, suggesting they felt less sensitivity around reporting. The impact of adjusting for individual-level characteristics was small; adjusted and unadjusted odds ratios for almost all contextual measures were broadly similar. Results describing variation in discordant reports by individual-level sociodemographic characteristics are available in supplementary Table S1.

## Discussion

This study is the first to explore state-level correlates of the substantial abortion underreporting found in the NSFG; however, we find no evidence that state-level measures of abortion stigma drive variation in underreporting as compared to external counts. At the aggregate level, there were no significant differences in reporting by any of our state-level measures; instead, abortion reporting was uniformly poor.

While we cannot assess the completeness of men’s reports using external data, at the individual-level we find similar levels of discordancy between ACASI and FTF reports among women and men. Regardless of gender, about 40% of respondents who report an abortion in the ACASI did not fully report in the FTF interview. Men’s increased reporting in the ACASI as compared to the less private FTF interview suggests that they also experience abortion as a sensitive behavior. This has serious implications for research using men’s abortion reports, and may challenge the theoretical construction of abortion stigma, which has generally conceptualized it as being rooted in ideals of womanhood. The identification of underreporting among men is a new contribution of this research and highlights potential similarities in men’s and women’s perception of abortion stigma and its impact on reporting in social surveys. This complements prior work in this area, which has found that men who disclose abortions to others are no more likely to receive positive or mixed reactions than women (Cowan, 2017).

Among women, at the individual level, for some indicators there was evidence that improvements in abortion reporting in ACASI as compared to the FTF interview was larger in states with more stigma. This suggests that the additional privacy and confidentiality afforded by ACASI was of greater importance to women living in states where they were exposed to greater structural stigma. Where abortion was less visible (through fewer clinics) or faced more negative public opinion, women were more likely to conceal their abortion from the survey interviewer. These findings further the conclusion of Lindberg and Scott (2018) that “the usefulness of ACASI varies in relationship to the social context and its relative effectiveness should not be assumed to be static.” The other three structural measures we tested showed no association with women’s reporting across survey modes, and none of the measures were significantly associated with the patterns of men’s reporting. A key limitation of the individual-level analysis for both genders is that it misses abortions that are not reported in either mode. Still, prior research interprets differences in reporting between modes as an indicator of the sensitivity of abortion (Peytchev et al., 2010).

Our study has several other limitations. Because of restrictions on what external data could be merged into the restricted-use NSFG data files, we recoded our continuous stigma measures into broad categories, which could have led to a loss of power It also is possible that the measures of state-level structural stigma that we selected were inadequate proxies for the true variation in abortion stigma that people experience based on where they live.^5^ Ideally, abortion stigma would be measured using validated scales; some prior work in this area has embedded these scales in national surveys, but not with sample sizes or survey designs that permitted stable estimation at subnational levels (Hanschmidt et al., 2016). In addition, the state may be the wrong geographical level of influence. We could not assess stigma’s impact on reporting at lower levels of geography because of the lack of corresponding external counts, but this seems like a fruitful avenue for future work.

The quality of abortion reporting in the NSFG for both women and men is poor; regardless of where in the country they live, they do not fully report their abortion experiences. The limited variation in the completeness of reporting across the state-level measures of stigma does not mean that abortion stigma is not prevalent in the USA; high levels of stigma surrounding abortion experiences have been extensively documented (Hanschmidt et al., 2016). The overall high levels of abortion underreporting that we find in the NSFG may themselves be considered a marker of stigma, as is the improved reporting with ACASI. Overall, the impact of this stigma on data quality is extensive; even data from states with the least evidence of structural stigma are incomplete and flawed. Improved approaches to asking sensitive survey questions are needed, and focusing on designing new questions, improved training of interviewers, or adaptation of computer-based survey modes may prove fruitful. More directly, reducing abortion stigma would improve not only survey data, but individuals’ lives and well-being.

## Supplementary Material

Appendix Table S1

## Figures and Tables

**Table 1:** State-level groupings by measures of structural stigma and weighted percent distribution of respondents in each area, by gender, pooled NSFG 2006–2015

Measure^b^	Number of states^a^	Percent distribution of respondents	
		Male (%)	Female (%)
Abortion laws			
Hostile (4–10 restrictions)	26	58	59
Middle-ground (2–3)	11	13	13
Supportive (0–1)	14	29	28
Abortion rate^c^ (per 1000 women aged 15–44)			
Low (6–10 abortions per 1000)	17	15	15
Middle (11–16)	17	34	34
High (17–33)	17	51	50
Number of abortion clinics^d^ (per 100,000 women aged 15–44)			
Low (2–7 clinics per 100,000)	17	26	27
Middle (7–17)	17	40	40
High (19–42)	17	34	33
Proportion of population who believes abortion should be legal in “all or most cases”			
Low (35–48%)	17	27	29
Middle (48–56%)	17	40	39
High (57–74%)	17	32	32
Proportion of population who says that religion is “very important” in their lives			
Low (32–47%)	17	32	32
Middle (48–56%)	17	34	33
High (56–77%)	17	34	35

a Numbers represent the categorization of each of the 50 states + DC, not the number of states represented in the NSFG sample

b Ranges shown in parentheses may overlap due to rounding

c By state of residence

d Clinics defined as facilities providing 400 or more abortions per year

**Table 2:** Results from logistic regressions predicting discordant reporting in the FTF and ACASI mode from selected state-level characteristics among women aged 15–44 reporting abortions in the ACASI mode, pooled NSFG 2006–2015

Measure	% Discordant	OR	*p*-value	AOR^c^	*p*-value
Total	0.41				
Abortion laws					
Hostile	0.42	1.00		1.00	
Middle-ground	0.34	0.70	0.28	0.78	0.47
Supportive	0.41	0.96	0.80	0.97	0.88
Abortion rate^a^					
Low	0.49	1.00		1.00	
Middle	0.44	0.80	0.47	0.74	0.40
High	0.38	0.63	0.13	0.57	0.11
Number of abortion clinics^b^					
Low	0.53	1.00		1.00	
Middle	0.36	0.50	0.00	0.50	0.01
High	0.39	0.58	0.01	0.57	0.02
Proportion of population who believes abortion should be legal in “all or most cases”					
Low	0.51	1.00		1.00	
Middle	0.38	0.58	0.01	0.66	0.06
High	0.39	0.60	0.01	0.66	0.04
Proportion of population who says that religion is “very important” in their lives					
Low	0.40	1.00		1.00	
Middle	0.37	0.91	0.67	0.90	0.65
High	0.47	1.36	0.13	1.30	0.23

*OR* odds ratio, *AOR* adjusted odds ratio

a By state of residence

b Clinics defined as facilities providing 400 or more abortions per year

c Each stigma measure tested in separate model adjusted for age, race, income level, union status, parity, urban/rural locality, nativity, and importance of religious life

**Table 3:** Results from logistic regressions predicting discordant reporting in the FTF and ACASI mode from selected state-level characteristics, among men aged 15–44 reporting abortions in the ACASI mode, pooled NSFG 2006–2015

Measure	% Discordant	OR	*p*-value	AOR^c^	*p*-value
Total	0.39				
Abortion laws					
Hostile	0.41	1.00		1.00	
Middle-ground	0.34	0.75	0.12	0.92	0.62
Supportive	0.39	0.94	0.65	0.90	0.54
Abortion rate^a^					
Low	0.36	1.00		1.00	
Middle	0.40	1.19	0.40	1.03	0.88
High	0.40	1.19	0.36	0.96	0.81
Number of abortion clinics^b^					
Low	0.40	1.00		1.00	
Middle	0.38	0.93	0.66	0.79	0.16
High	0.40	1.01	0.97	0.79	0.19
Proportion of population who believes abortion should be legal in “all or most cases”					
Low	0.43	1.00		1.00	
Middle	0.37	0.75	0.07	0.80	0.14
High	0.39	0.85	0.32	0.86	0.38
Proportion of population who says that religion is “very important” is in their lives					
Low	0.37	1.00		1.00	
Middle	0.38	1.03	0.86	1.24	0.23
High	0.43	1.29	0.10	1.26	0.15

*OR* odds ratio, *AOR* adjusted odds ratio

a By state of residence

b Clinics defined as facilities providing 400 or more abortions per year

c Each stigma measure tested in separate model adjusted for age, race, income level, union status, urban/rural locality, previous births, nativity, and importance of religious life

## Data Availability

Public use data files for the NSFG are available at https://www.cdc.gov/nchs/nsfg/index.htm. Analyses in this manuscript use restricted contextual data only available through the NCHS Research Data Center.

## Footnotes

1 All NSFG respondents self-report their current gender at the time of interview, which determines the questionnaire they are routed into.

2 Because there are no external counts of abortion for men, this aggregate level analysis could not be done for male NSFG respondents.

3 A small number of respondents reported more abortions in the FTF than the ACASI (N = 32 for women, N = 26 for men); these cases were coded as concordant for the purpose of this analysis; we also dropped from all analyses a small number of respondents with reports in the ACASI of more than 30 abortions in the past five years (N = 2 for women, N = 1 for men).

4 We were unable to use multilevel models for this analysis as they would require split sampling weights for each level, which the NSFG does not make available.

5 These state groupings did not mask larger regional differences; we found no differences in abortion reporting by a four-category geographic region measure.
