# Supplementary material for: State-Level Variation in Abortion Stigma and Women and Men’s Abortion Underreporting in the USA
Source: Popul Res Policy Rev. Author manuscript; Available in PMC 2021 Dec 1. (PMC8547795; doi:10.1007/s11113-021-09657-4)
Supplement: Appendix Table S1 [file NIHMS1737501-supplement-Appendix_Table_S1.pdf]

Appendix Table S1. Proportion of respondents with discordant reporting in the FTF and ACASI mode according to individual level characteristics, among respondents aged 15-44 reporting abortions in the ACASI mode, stratified by gender, pooled NSFG 2006-2015.

	Male		Female	
Measure	% discordant	p-value	% discordant	p-value
**Total**	0.39		0.41	
**Age at interview**				
15-24	0.46	0.08	0.26	0.01
25-29 *(ref)*	0.37		0.40	
30-34	0.37	0.86	0.44	0.51
35 and older	0.39	0.62	0.60	0.00
**Race/ethnicity**				
White, non-Hispanic *(ref)*	0.26		0.36	
Black, non-Hispanic	0.45	0.00	0.40	0.40
Other, non-Hispanic	0.47	0.00	0.42	0.50
Hispanic	0.57	0.00	0.50	0.01
**Poverty status**				
<100% *(ref)*	0.56		0.48	
100-199%	0.39	0.00	0.39	0.03
200+%	0.32	0.00	0.34	0.02
**Union status**				
Married *(ref)*	0.44		0.56	
Cohabiting	0.43	0.87	0.38	0.01
Not in union	0.33	0.00	0.35	0.00
**Residence**				
Urban *(ref)*	0.42		0.38	
Suburban/Rural	0.38	0.21	0.44	0.15
**Parity**				
0 *(ref)*	0.32		0.35	
1+	0.43	0.00	0.45	0.04
**Nativity status**				
U.S.-born *(ref)*	0.32		0.35	
Foreign-born	0.68	0.00	0.64	0.00
**How important religion is in R's daily life**				
Somewhat or not important *(ref)*	0.34		0.34	
Very important	0.50	0.00	0.52	0.00
